# Enhancing mathematics teachers’ quality through Lesson Study

**DOI:** 10.1186/s40064-016-3215-0

**Published:** 2016-09-15

**Authors:** Laila S. Lomibao

**Affiliations:** Department of Mathematics Education, College of Policy Studies, Education and Management, Mindanao University of Science and Technology, Claro M. Recto Avenue, Lapasan, Cagayan de Oro City, 9000 Misamis Oriental Philippines

**Keywords:** Lesson Study, Mathematics teachers’ quality, Teachers’ professional development

## Abstract

The efficiency and effectivity of the learning experience is dependent on the teacher quality, thus, enhancing teacher’s quality is vital in improving the students learning outcome. Since, the usual top-down one-shot cascading model practice for teachers’ professional development in Philippines has been observed to have much information dilution, and the Southeast Asian Ministers of Education Organization demanded the need to develop mathematics teachers’ quality standards through the Southeast Asia Regional Standards for Mathematics Teachers (SEARS-MT), thus, an intensive, ongoing professional development model should be provided to teachers. This study was undertaken to determine the impact of Lesson Study on Bulua National High School mathematics teachers’ quality level in terms of SEARS-MT dimensions. A mixed method of quantitative–qualitative research design was employed. Results of the analysis revealed that Lesson Study effectively enhanced mathematics teachers’ quality and promoted teachers professional development. Teachers positively perceived Lesson Study to be beneficial for them to become a better mathematics teacher.

## Background

Lesson planning in the Philippines teaching practice has been an isolated work. Designing and preparing a lesson is usually an individual teacher’s task. It is an individual teacher’s responsibility to decide how the lesson will be delivered, what materials to be used and how students be evaluated. This indicates that the efficiency and effectivity of the learning experience is dependent on the teacher’s ability and quality. According to Nye et al. (2004), Chetty et al. (2014), teachers have substantial impacts on their students’ academic and life-long success. Clements et al. (2013) agreed and stated that teacher as an aspect of mathematics instructional environment is related to student achievement. Wenglinsky (2001) added that teacher quality has significant effect on student achievement. He particularly stressed that high-quality professional development focusing on higher-order thinking skills and diversity issues does appear to strongly influence classroom practice. As a matter of fact, teacher quality and classroom practice have an equal to or exceeding effect on student achievement than socioeconomic status.

Hence, enhancing teacher’s quality is vital in improving the students learning outcome. The teacher’s academic advancement and professional upgrading activities contribute to an enjoyable and productive teaching (Bayocot 2014). And to realize this, there should be an intensive, ongoing professional development model provided to teachers. A development model that is connected to teaching practices focused on students learning and addresses the teaching of specific curriculum content aligned to school improvement priorities and goals, and which also build strong working relationships among teachers (Darling-Hammond et al. 2009).

The Southeast Asian Ministers of Education Organization (SEAMEO) demanded the need to develop mathematics teachers’ quality standards that would facilitate students’ mathematics learning outcomes. SEAMEO Regional Centre for Education in Science and Mathematics (Tahir and Thien 2013) has outlined the Southeast Asia Regional Standards for Mathematics Teachers (SEARS-MT). This was conceptualized to provide a cogent standard in mathematics teachers quality development which can be used as benchmark for monitoring and improving teacher quality. This consists of four dimensions namely the professional knowledge, professional teaching, personal and professional attributes and professional community.

On the other hand, Japan implemented Lesson Study (LS) as a fundamental driver for mathematics teacher development. LS has been the primary mechanism of professional development for both prospective teachers and practicing teachers since the Japanese public education system started (Lewis 2000; Lewis and Tsuchida 1998; Makinae 2010; Murata and Takahashi 2002; Takahashi 2000; Takahashi and Yoshida 2004; Yoshida 1999a). It is a school-based collaborative activity for teachers mainly characterized by a continuous cycle of meticulous planning, prudent and mindful demonstrating, and perceptive improving of a lesson. It is a wide scope, on-site professional development process that involves a small group of teachers. The teachers are of different levels of ability but with interest in working collaboratively, with specific objectives for lesson planning, to carry out the planned and researched lesson (Fong 2015). It is a process in which teachers progressively strive to improve their teaching methods by working with other teachers to examine and critique one another’s teaching techniques (Isoda et al. 2007). Japanese teachers use LS as the core process of professional learning to continually improve the quality of educational experiences they provide their students (Iverson and Yoshida 2005). According to Takahashi (2015) LS is not just a “nice to have, but a must have”. He stressed that LS provides opportunity for classroom teachers to work collaboratively to seek effective implementation of new ideas, rather than struggle in isolation to understand how the ideas look in his/her own classroom. He elaborated that LS provides access to outside experts, the knowledgeable others, so that each teacher can understand new ideas for improving teaching and learning with concrete examples. He added that LS as a fundamental driver for professional development permits teachers to learn not only new ideas for improving teaching and learning but also helps them to develop expertise.

With these aforementioned views, this paper aims to assess Bulua National High School mathematics teachers’ quality level in terms of the SEARS-MT dimensions, and explore the use of LS to enhance mathematics teachers’ quality and to promote teachers professional development (TPD). Also, this paper aims to determine the teachers’ perception on the use of LS for TPD.

### An overview of TPD and Lesson Study in the Philippines

Professional development for teachers in the Philippines were usually done through a school or division level in-service trainings and seminars. These trainings and seminars were an echo seminar conducted by the participants trained in the regional or national level trainings. Teacher-participants in the school and division level acted as audience for the talks and demonstrations of the speakers or resource person—who were the participants in the regional and national level trainings. The training or seminar usually span for 2–5 days in maximum, twice a year, during midyear break and summer break.

Bentillo et al. (2003) called this professional development for teachers as cascading model, where the training moves from the national, regional, division, then school level with decreasing duration at each lower level. He added that this training is often used to implement changes on a nationwide scale such as the curriculum reform and promotion of the new approach or learning strategies and the training content is decided at the central level. However, commented that there was much dilution in using this top-down one-shot model.

Ulep (2006) also identified cluster-based training as another model used for professional development in the Philippines. This model involves teachers from several schools attending the same training program conducted by invited subject specialists as trainers. The content is determined by the master teachers, the department coordinator of the schools in consultation with the teachers. She claimed that dilution may be avoided in this model, but she also commented that if the trainers are not fully aware of the schools’ situations, hence, the relevance of the training may not be well appreciated by the teachers.

Gutierez (2015) documented in her study on the year-long professional development of the teachers in the Philippines. She observed that experts tried to build each teacher’s capacities within a set of concrete activities including lecture sessions, workshops, collaborative lesson planning, classroom observations, and post-lesson reflections and discussions. This was confirmed by Loucks-Horsley et al. (1998) claim, that in the Philippines, most of the implemented professional development efforts are designed to model inquiry teaching and actively engage teachers as learners rather than as information gatherers, for them to influence their instructional practices, and to enhance their knowledge and skills. Thus, she further stressed the strong need for teachers to engage in sustainable professional development, to provide teachers with sustainable support as they face new and current challenges, and at the same time foster the development of learning communities where they provide instructional support to each other. As she characterized the teachers’ insights, she found out that the teachers’ analyses of their instructional practices deepen as they engage continuously in collaborative and constructive self-assessment and discussions.

On the other hand, the Collaborative Lesson Research and Development (CLRD) Project of the University of the Philippines National Institute for Science and Mathematics Development (UP NISMED) introduced LS to mathematics and science teachers and administrators of elementary and secondary schools and used it to promote teaching mathematics through problem solving and teaching science through inquiry in 2006. The project spanned for three years (2006–2009) in four (4) schools in the National Capital Region (NCR). A total of about 60 teachers and 10 UP NISMED Mathematics staff were involved. In 2010, the project extended to other four schools in the NCR: two schools in Pasig City, the Santa Lucia High School in all four year levels of mathematics subject and Rizal High School on science subjects: Earth & Environmental Science, Biology, Chemistry; and two schools in Quezon City, the Commonwealth Elementary School and North Fairview High School. A total of 52 teachers and 29 UP NISMED science and mathematics staff were involved. Seminar workshops were also conducted to 20 teachers of Ligao National High School (LGNHS) in Region 5 last May, 2011. This was the first case that teachers from a school outside NCR learned about LS. In September 2011, UP NISMED staff followed the teachers of LGNHS as they did LS on their own with the staff serving as knowledgeable other. In January to February, 2012 a total of 40 science and mathematics teachers of Nueva Ecija High School in Region 3 and 13 UP NISMED staff did LS. The project report showed that teachers and students positively appreciated the effects of LS, for the opportunities created for them to think independently and deeply as they did the research lessons. Teachers found students to be more responsive and interested to learn science and mathematics as well as they acknowledged that LS enhanced their content and pedagogical content knowledge through sharing of ideas during planning and post-lesson discussions and observation of classes. Teachers appreciated the trust, openness, unity, and camaraderie that they experience which give them confidence in teaching the research lesson (Ulep and Obille Jr. 2013). They also reported that though LS is widely adopted by other countries such as our neighboring Asian countries and the US, LS in the Philippines is not extensively disseminated yet.

However, Ronda (2013) cited that all the teachers in the LS group commented that, “they used the strategies learned during the Lesson Study to teach the succeeding topics but went back to their old way of teachings”. Ronda stressed that there is a need to find ways of sustaining teachers in their effort to improve their own teaching and further pointed out that, though the innovation of LS was effectively practiced, it did not result in the level of adaptation found in the United States. Ebaeguin and Stephens (2015) confirmed this in the result of his investigation on the adaptation and cultural transition of the implementation of LS in non-Japanese context, the Philippines. He found out that it is necessary to consider the existing habits and values teachers have in a particular school that may support the implementation of LS because if not taken into consideration possible cultural barriers in transferring and implementing LS into a different national context may lead to delay in reaping its benefits, if not wastage of time, effort and resources of its stakeholders. He also stressed that fidelity approach should be taken into consideration by asking what skills and knowledge teachers need in order to implement LS faithfully. Nonetheless, Laynesa et al. (2013) also concluded in their reports that the positive response of the teachers under study signifies that the approach is possible in the Philippines, that eventually, teachers will regard the practice as a normal or ordinary occurrence. However, they are quick to point out that LS is not yet widely in use in the Philippines and initial barriers to its implementation included negative feelings experienced by the teachers at the onset. Nevertheless, Stigler and Hiebert (1999); Yoshida (1999a, b) have seen LS as a promising school-based TPD activity which can be used in the Philippines.

### Integrating LS and SEARS-MT for TPD

This study is anchored on the concept of *jugyokenkyu,* which came from the Japanese word *jugyo* (*lesson)* and *kenkyu* (*study)* or simply Lesson Study. Lesson Study originally a Japanese practice of enhancing teaching practice, wherein teachers conduct a systematic inquiry into their pedagogical practice by closely examining their lesson (Fernandez 2002).

In LS, a group of teachers with specific objectives for lesson planning meet together and investigate a particular lesson in four-stage cycle. The group is composed of the teacher-participants and the Knowledgeable One (KO)—a master in content and pedagogical knowledge. The first stage is the planning of the Research Lesson (RL)—the particular lesson to be studied, where teachers collaboratively examine the RL by studying the particular content knowledge together, decide how the required competencies to be mastered by student will be attained, plan how the RL should be delivered, what particular strategy will be best used in the particular RL, prepare the necessary instructional materials such as ICT models or computer applications and programs, and creatively design a lesson plan to impact students’ achievement. In this stage teachers of different levels of ability can interact with each other with the KO as the supervisor and facilitator of the discussion and learning. In this process the more advanced peers can help the less advanced members.

This practice is in accord with the SEARS-MT dimension 1: Professional Knowledge, which stipulates that teachers should have knowledge in understanding of fundamental mathematical ideas, principles, and teaching approaches, knowledge of the characteristics of students and their implications for learning, knowledge of supporting creativity and the development of higher order thinking skills, and knowledge of ICT to model and solve problems.

This is also an important opportunity for mathematics teachers since a high demand of these skills are required from them with the implementation of the new K to 12 curriculum of the Department of Education (DepEd) that set changes in the framework of the mathematics learning in the Philippines. With this curriculum, teachers assigned in a year level will prepare themself to teach all contents (Number and Number Sense, Measurement, Geometry, Patterns & Algebra and Statistics and Probability) since the competencies are designed in spiral where concepts for a particular field is taught in increasing level of difficulty. Thus, studying a RL and designing a lesson plan together will enhance the teachers’ content and pedagogical knowledge.

The second stage of the LS is conducting the RL—implementing and observing the research lesson. The designed RL is taught by one teacher from the group and the other team members observe. Observers take notes of the significant events during delivery of the RL. Giving significant attention on the teacher-learners interaction, student–student discourse. Observing if the RL and the teacher implementing the RL have maximize and enhance discourse, encourage high order thinking skills, create an motivating learning environment and relation with the students, and provide students with adequate mathematical tasks and opportunity to analyze and reflect upon their own mathematical thinking. All these activities supports SEARS-MT dimension 2: Professional Teaching.

LS stage 3 provides opportunity for teachers to develop qualities described in SEARS-MT dimension 3. The group convenes to conduct a post-lesson discussion and reflect on the observed lesson. Observers and the implementer alike can see and learn how students’ responded in class and to the lesson. Teachers will learn more about their students and identify their skills and capacity. Teachers can gain insights into the teaching–learning process. Furthermore, teachers can improve personal attributes on how to assist students to engage, to appreciate, to value their learning and to achieve their potential. Teachers can develop to exhibit care and respect for their students. The teacher-participants of the LS can also enrich professional and moral behavior towards colleagues, as they considered discussion and observations as constructive criticism to advance and value continual improvement of personal professional development.

**Fig. 1:**
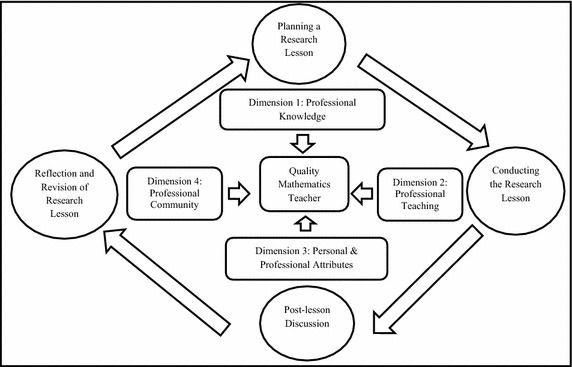
Integrating LS and SEARS-MT to promote TPD

Reflecting on what have been learned from the post-discussion (LS stage 4), revison of the RL follows to refine the it. Integrating important points discussed as the result of the post-discussion to improve RL. The refined RL will be implemented and observed again. Then another cycle will take place until a more refined and better RL for a particular topic is produced. This activity will show the teachers commitment to engage with the school and community to promote mathematics learning. In addition, reporting and sharing thoughts about the outcomes of the RL through action research and articles may contribute helpful knowledge to the profissional communities both at school and outside the schools. These qualities stipulated in dimension 4 of SEARS-MT. Figure 1 summarizes the interplay of LS and SEARS-MT dimensions.

The principles of integrating Lesson Study and SEARS-MT is also grounded on Lave’s (1988) social and situated theory. He argued that learning as it normally occurs is a function of the activity, context and culture in which it occurs. He stressed that social interaction is a critical component of situated learning—learners become involved in a “community of practice” which embodies certain beliefs and behaviors to be acquired. As the beginner or newcomer moves from the periphery of this community to its center, they become more active and engaged within the culture and hence assume the role of expert or old-timer. These coincide with the idea of LS which considered learning as a social and situated process, where teachers’ social interact and communicate to plan and learn together and is situated in their own classroom as the best venue for them to learn and improve their teaching practices. These ideas are what Lave and Wenger (1990) call the process of “legitimate peripheral participation.”

This study is also anchored on Schon’s (1983) notion of *reflection in action* and *reflection on action*. The process of LS can be linked with these two concepts of reflection, as `*reflecting while doing*’ when teachers gathered and plan for the research lesson and while observing the teacher-implementer during class demonstration, and `*reflecting after doing’* when teachers meet again for the post-discussion. When the observation is complete the teachers can reflect on, analyse and evaluate the learning and teaching. This post- action reflection then informs the subsequent planning and preparation leading to a cycle of continuing improvement. Thus, it is vital that these learning experiences are recorded in journals and discussed with mentors and fellow trainees.

## Methods

The study employed a mixed method of quantitative–qualitative research design. The quantitative part was focused to determine the influence of the LS on teachers. T test was used to determine if Lesson Study have significant impact on the teachers’ professional knowledge, professional teaching, personal and professional attributes, and professional communities. The qualitative part is focused on getting teachers’ perception on LS as TPD through a survey questionnaire and interview for triangulation.

A survey questionnaire formulated based on the attributes stipulated in SEARS-MT was the main instrument of the study. In determining the teachers’ quality level on each domain, a Likert-scale was utilized to give value to the teachers’ responses. To rate the teachers’ quality level, a highest score of 5 is given to an expert teacher where all attributes are manifested in a given indicator, 4 for master has 4 out of 5 attributes are manifested, 3 for practitioner where 3 attributes are manifested and 1 for novice with only one or no attribute manifested. In analyzing the data gathered, mean and standard deviation were computed. The second instrument used was a survey questionnaire adopted from Hj Suhaili and Shahrill (2015) with slight modification to gather insights on the teachers’ perception of Lesson Study and as guide questions for the interview to verify their responses.

Five (5) mathematics teachers of Bulua National High School were the participants of the study. The teachers were all teaching grade 10 mathematics. One teachers acted as the team leader, she was the chairman for the grade level, an experienced teacher in terms of years of service and had units in master’s degree in educational management. One teacher has been teaching mathematics for more than 20 years, but did not have any graduate units. The other three teachers were composed of less experienced teachers with less than five years teaching experience. Two of them had graduate units in mathematics teaching and the remaining one have no graduate units. The researcher acted as the knowledgeable one (KO).

The KO introduced Lesson Study to the teacher-participants, discussed the nature of LS, and oriented them on how it will be conducted. After the teachers understand the concept of LS and agreed to engage in the study, they then took the pre-assessment, to determine the teachers’ quality level in terms of SEARS-MT dimensions. After the pre-assessment, the team arranged for the schedule of activities including the schedule of regular meetings. Post-assessment were done after two grading periods. Table 1 below provides the overview of the LS conducted.

**Table 1:** Overview of the conduct of Lesson Study in designing one research study

Lesson Study stage	Specific activity
Cycle 1	
Session 1	
Planning a RL	Selected teaching topics
	Selected teaching class and teacher implementer of RL
	Identified specific mathematics content and competencies to be taught
	Formulated teaching objectives
	Determined prerequisites knowledge and skills
	Decided and planned for appropriate teaching strategy to be employed.
	Designed students’ classroom activity
	Prepared learning materials
Session 2	
Conducting the RL	Teacher 1 (T1) implemented RL in the class
	Other teachers and KO observed the RL implementation in class
	Video recording of the class
Session 3	
Post-lesson discussion	Reviewed the class implementation of RL through the video recorded
	Discussed the result of the observation
Reflection and revision of RL	Analyzed results of the post-discussion and reflected on the conducted RL and activity implemented in class
	Revised RL
Cycle 2	
Session 4	
Planning for implementation of the revised RL	Planned revised RL integrating significant points noted for revision
Session 5	
Conducting revised RL	Teacher 2 (T2) implemented RL in the class
	Other teachers and KO observed the RL implementation in class
	Video recording of the class
Session 6	
Post-lesson discussion	Reviewed the class implementation of RL through the video recorded
	Discussed the result of the observation
Reflection and revision of RL	Analyzed results of the post-discussion and reflected on the conducted RL and activity implemented in class
	Revised RL for refinement

The RL covers topics in the second and third grading periods of the grade 10 mathematics curriculum which includes the topics on polynomial functions particularly, polynomials using long division and synthetic division, remainder theorem and the factor theorem, polynomial equations, rational root theorem, polynomial equations, graphs polynomial functions, problem solving involving polynomial functions; and topics on circles including chords, arcs, central angles, inscribed angles, secants, tangents, segments, sectors of a circle, and geometric figures on the coordinate plane.

## Results and discussion

Table 2 compares the means of the pre-assessment and post-assessment of the level of teachers’ quality in terms of the four dimensions of the SEARS-MT. It reveals a positive mean difference between the post-assessment and the pre-assessment. The tables show that the teachers’ quality increases from a practitioner level to master level. This indicates a positive impact of LS in improving the mathematics teachers’ quality in terms of the criteria set by the SEARS-MT.

**Table 2:** Descriptive statistics and paired t test of teachers’ quality level in terms of SEARS-MT dimensions

Domains	Pre-assessment		Post-assessment		Mean Diff.	t value	p value
	$\bar{X}_{1}$	SD	$\bar{X}_{2}$	SD	$\bar{d}$		*α* = 0.05.level of significance
Domain 1: professional knowledge	2.6		3.8				
Knowledge of mathematics	2.4		3.7				
Knowledge of student’s learning Mathematics	2.2		3.5				
Knowledge of intellectual quality	2.4		2.9				
	2.40	0.16	3.48	0.40	1.08	2.78	0.0114
Domain 2: professional teaching							
Mathematical tasks and discourse	2.4		3.6				
Planning for learning process	2.2		3.5				
Implementing teaching strategies	2.2		3.9				
Monitoring, assessment and evaluation	2.4		3.9				
Use of ICT	2.2		3.3				
	2.28	0.11	3.64	0.26	1.36	5.65	0.0002
Domain 3: Personal and professional attributes							
Personal attributes	3.6		4.1				
Personal professional development	2.0		4.0				
Personal responsibilities towards community	2.6		3.2				
	2.73	0.81	3.77	0.49	1.04	1.23	0.1664
Domain 4: Professional communities							
Professional ethics	3.2		4.7				
Professional communities at schools	2.4		3.7				
Professional communities outside schools	2.0		3.0				
	2.53	0.61	3.8	0.85	1.27	5.03	0.0129
Overall	2.45	0.43	3.65	0.46	1.20	2.84	0.0001
	Practitioner		Master				

Weight	Mean range	Descriptive level
5	4.5–5.0	Expert
4	3.5–4.49	Master
3	2.5–3.49	Practitioner
2	1.5–2.49	Apprentice
1	1.0–1.49	Novice

Teachers in practitioner level have demonstrated at least two out of four indicators in each components of the four dimensions. They generally have knowledge about the mathematics content and mastery in procedural knowledge, and showed understanding of the new K to 12 curriculum. Though they used questions effectively to scaffold instruction and always used wait time and questioning effectively to diagnosed problems with learning and improved instruction, they used generic self-evaluation strategies or tools which did not explicitly show improvement on students’ self-learning and showed a limited sensitivity to student affect, the teacher tailored feedback for only a few students because the teacher gave more emphasis on teaching the specific content. Also they have showed basic knowledge of ICT but did not use it in class. In addition, practitioner teachers appreciated the beauty and importance of mathematics and highly passionate about teaching mathematics. They did not engage also any organization in related to mathematics except in their subject area department. While teachers in master level have shown three out of four indicators in each components of the four SEARS-MT dimensions. They demonstrated understanding of the nature and scope of mathematics content to be taught throughout the curriculum and its relevance to teaching, have the ability to explain fundamental principles in terms of precision, definition, reasoning, connections, coherence and purposefulness as well as procedural fluency. They generally used questions effectively to scaffold instruction and always used wait time and questioning effectively to diagnose problems with learning and improve instruction. They provided varied strategies for students to use for self-evaluation during instruction in an effort to regulate and improve the students’ self-learning by utilizing tools such as the use checklists, rubrics, drawings, a self-assessment inventory, journaling, and/or reflection statements. Also, utilized technology in class to increase visual, graphing and computing efficiency. Furthermore, master teachers exhibit enthusiasm and confidence for both mathematics and learning. Continuously enrich and upgrade knowledge and skills pertaining to mathematics and mathematics teaching and enrich educational experience by affiliating with professional organizations and community and promote mathematics in school and outside school.

The t-test yielded a computed probability-value of less than 0.05 level of significance for the three dimensions: personal knowledge (0.0114 < 0.05), professional teaching (0.0002 < 0.05) and professional communities (0.0129 < 0.05). This led to the researcher to reject the null hypothesis. This implies that there is a significant difference in the pre-assessment and post-assessment results. This means that the implementation of LS have contributed to the increase the teachers quality particularly in the teachers professional knowledge, professional teaching and professional community. However t test further revealed LS does not have a significant effect on the teachers’ personal and professional attributes with computed p value of 0.1664 which is greater than the 0.05 level of significance. This maybe because teachers were enthusiast and confidence on mathematics and learning mathematics even before LS was introduced. They already appreciated and understood the importance of mathematics. Thus, there were no significant change can be observed before and after on the introduction of LS on the dimension of personal and professional attributes.

In general, it can be gleaned from the table that the overall p value of 0.0001, is less than the 0.05 level of significance. This means that LS has a significance impact on the teachers’ personal development. This indicates that LS had positive impact to the teachers’ professional development in terms of enhancing mathematics teachers’ professional knowledge and professional teaching, and broaden professional communities. The results confirm the findings of Iverson and Yoshida (2005), and Takahashi (2015) that emphasis that LS is not just a nice to have but a must have.

Table 3 shows the percentage distribution of the teachers’ response to each nineteen statements on their perception on impact of Lesson Study. Generally, the teachers involved in the LS conducted, had strongly agreed that LS has positively influenced their professional development. In particular, all of them (100 %) responded that planning a research lesson together have broadened their knowledge of the mathematics content, helped them understand more on students’ way of thinking and learning mathematics concept, and broadened their knowledge of mathematics teaching ideas and pedagogy, which all constitute the dimension on professional knowledge of SEARS-MT.

**Table 3:** Teacher’s perception on the impact of Lesson Study Adopted from Hj Suhaili and Shahrill (2015)

Statements	Percentage				
	SD	D	U	A	SA
1. Planning together broadened my knowledge of the mathematics content/subject matter					100
2. Planning and preparing to teach the topic we have chosen caused me to engage in mathematical reasoning and problem solving				80	20
3. Planning together helped me to be aware of the new mathematics curriculum and have a deeper understanding of the K to 12 Mathematics Curriculum				80	20
4. Planning together helped me understand more on students’ way of thinking and learning mathematics concept					100
5. Planning in a group broadened my knowledge of mathematics teaching ideas and pedagogy					100
6. The collaborative lesson planning is beneficial for me in order for me to be a better mathematics teacher					100
7. Observing and analyzing others’ lesson helped me think more deeply about mine					100
8. Teaching and observing the research lessons made me more critical in choosing the right teaching activities that help students to understand and think mathematically					100
9. The reflective comments made me more aware of my general weaknesses and strengths of my own mathematics teaching				20	80
10. The comments and feedback can help me to be a better mathematics teacher					100
11. My teaching has improved after taking part in the Lesson Study				60	40
12. I have become more conscious and sensitive to students’ learning needs and difficulties and have a deeper understanding of how students learn				80	20
13. The Lesson Study contributes to teachers’ professional development					100
14. The Lesson Study has improved my understanding of students’ learning				80	20
15. I learnt a better way to teach the topic				40	60
16. I learnt that it is important to provide activities that encourage students to think critically and creatively				20	80
17. I learnt that Lesson Study can be implemented and are sustained in my school				60	40
18. The experiences and knowledge I gained during the Lesson Study is very valuable and important in order to make me a better mathematics teacher					100
19. I am willing to take part in the Lesson Study					100
Mean percentage				24.21	75.79

*SD* strongly disagree, *D* disagree, *U* undecided, *A* agree, *SA* strongly agree

The teachers also have positive response on giving feedback about their teaching during the actual observation as well as in the video critiquing of the implementation of RS. They unanimously strongly agreed that observing and analyzing others’ lesson helped them reflect more deeply about their own teaching practice, made them more critical in choosing the right teaching activities that help will students to understand and think mathematically, and that the comments and feedback can help them to be a better mathematics teacher, which are components specified in the dimension on professional teaching.

Furthermore, they all strongly agreed that LS contributes to TPD. All of them strongly agreed that collaboratively working together is beneficial for them to become a better mathematics teacher. They strongly stressed that the experiences and knowledge they gained during the Lesson Study is very valuable and important in order to make me a better mathematics teacher. Thus, 100 % expressed that they are willing to take part in the Lesson Study.

## Conclusion and recommendation

Based on the findings of the study, the researcher concludes that for this group of teachers, Lesson Study effectively enhance mathematics teachers’ quality and promote teachers professional development. Hence, the researcher recommends the use of Lesson Study improve the mathematics teachers’ quality. Similar studies may be conducted to a wider scope using different population to promote the generalizability of the results.

## References

[CR1] Bayocot A (2014) Philippine Public School Teachers Association country report Philippines. Topic: Balancing the teaching activities in the classroom with crucial professional upgrading activities for teachers. The 30th ASEAN Council of Teachers Convention, Singapore

[CR2] Bentillo E, Carale L, Galvez E, Magno M, Pabellon J, Talisayon V, Tan M, Treyes R (2003). Teacher development Supervision of science and mathematics teaching.

[CR3] Chetty R, Friedman JN, Rockoff JE (2014). Measuring the impacts of teachers II: teacher value-added and student outcomes in adulthood. Am Econ Rev 104(9):2633–2679. In: Blazar D (2015) Effective teaching in elementary mathematics: Identifying classroom practices that support student achievement. Econ Educ Rev 48(2015)16–29. www.elsevier.com/locate/econedurev. Downloaded 20 April 2016

[CR4] Clements D, Agodini R, Harris B (2013) Instructional practices and student math achievement: correlations from a study of math curricula. National Center for Educational Evaluation and Regional Assistance. Institute of Education Sciences. http://ies.ed.gov/ncee/pubs/20134020/pdf/20134020.pdf. Accessed 20 Apr 2016

[CR5] Darling-Hammond L, Wei RC, Andree A, Richardson N, Orphanos S (2009) Professional learning in the learning profession: a status report on teacher development in the United States and abroad. National Staff Development Council. Stanford, CA. http://learningforward.org/docs/pdf/nsdcstudy2009.pdf

[CR6] Ebaeguin M, Stephens M (2015) Teacher professional development through Lesson Study: adaptation or cultural transition? In: 7th ICMI-East Asia regional conference on mathematics education. Cebu City, Philippines

[CR7] Fernandez C (2002). Learning from Japanese approaches to professional development. J Teach Educ.

[CR8] Fong MW (2015) Effects of Lesson Study incorporating phase-based instruction on grade seventh students’ geometry achievement. In: 7th ICMI-East Asia regional conference on mathematics education. Cebu City, Philippines

[CR9] Gutierez SB (2015). Collaborative professional learning: Implementing inquiry based teaching through Lesson Study. Issues Educ Res.

[CR10] Hj Suhaili A, Shahrill M (2015) A comparison study of Bruneian primary mathematics teachers’ perceived learning in Lesson Study. In: 7th ICMI-East Asia regional conference on mathematics education. Cebu City, Philippines

[CR11] Isoda M, Stephens M, Ohara Y, Miyakawa T (2007). Japanese Lesson Study in mathematics: its impact, diversity and potential for educational improvement.

[CR12] Iverson PW, Yoshida M (2005). Building our understanding of Lesson Study.

[CR13] Lave J (1988). Cognition in practice: mind, mathematics, and culture in everyday life.

[CR14] Lave J, Wenger E (1990). Situated learning: legitimate periperal participation.

[CR15] Laynesa E, Mirana A, Huemo E, Ruiz M, Nacario C (2013). Students’ performance in science in three selected schools using Lesson Study: a pilot study. NUE J Int Educ Coop.

[CR16] Lewis C (2000) Lesson Study: the core of Japanese professional development. Paper presented at the AERA annual meeting

[CR17] Lewis C, Tsuchida I (1998) A lesson is like a swiftly flowing. In: Takahashi A, (eds) Lesson Study: nice-to-have or must-have? 7th ICMI-East Asia regional conference on mathematics education. Cebu City, Philippines

[CR18] Loucks-Horsley S, Hewson PW, Love N, Stiles KE (1998). Designing professional development for teachers of science and mathematics.

[CR19] Makinae N (2010) The origin of Lesson Study in Japan. Paper presented at the 5th East Asia regional conference on mathematics education: In: Search of excellence in mathematics education, Tokyo

[CR20] Murata A, Takahashi A (2002) Vehicle to connect theory, research, and practice: How teacher thinking changes in district-level Lesson Study in Japan. In: Proceedings of the twenty-fourth annual meeting of North American chapter of the international group of the psychology of mathematics education, pp 1879–1888

[CR21] Nye B, Konstantopoulos S, Hedges LV (2004) How large are teacher effects? Educ Eval Policy Anal 26(3):237–257. In: Blazar D (2015) Effective teaching in elementary mathematics: Identifying classroom practices that support student achievement. Econ Educ Rev 48(2015):16–29. www.elsevier.com/locate/econedure. Downloaded 20 Apr 2016

[CR22] Ronda E (2013) Scaffolding teacher learning though Lesson Study. In: Ulep S, Punzalan A, Ferido M, Reyes R (eds) Lesson study: planning together, learning together. UPNISMED, Quezon City, pp 195–216. http://math4teaching.com/wp-contentluploads/2014/07/Ronda_Scaffolding-Teacher-Learning.pdf

[CR23] Schon D (1983) The reflective practitioner: How professionals think in action. Basic Books, New York. Cited by Dr. Mark L. Merickel. (1998). Reflective practice the reflective practitioner. http://oregonstate.edu/instruct/pte/module2/rp.htm. Downloaded 17 Aug 2016

[CR24] Stigler JW, Hiebert J (1999). The teaching gap: best ideas from the world’s teachers for improving education in the classroom.

[CR25] Tahir S, Thien LM (2013). Southeast Asia Regional Standards for Mathematics Teachers (SEARS-MT): setting the bar for the teachers.

[CR26] Takahashi A (2000). Current trends and issues in Lesson Study in Japan and the United States. J Jpn Soc Math Educ.

[CR27] Takahashi A (2015) Lesson Study: nice-to-have or must-have? In: 7th ICMI-East Asia regional conference on mathematics education. Cebu City, Philippines

[CR28] Takahashi A, Yoshida M (2004). How can we start Lesson Study? ideas for establishing Lesson Study communities. Teach Child Math.

[CR29] Ulep S (2006) The potential of Lesson Study in enabling teachers to implement in their classes what they have learned from a training program. University of the Philippines National Institute for Science and Mathematics Education Development (UP NISMED)

[CR30] Ulep S, Obille Jr E (2013) Report on the Lesson Study in science and mathematics education in the Philippines. University of the Philippines National Institute for Science and Mathematics Education Development (UP NISMED)

[CR31] Wenglinsky H (2001) https://www.ets.org/Media/Research/pdf/RR-01-19-Wenglinsky.pdf. Downloaded 20 Apr 2016

[CR32] Yoshida M (1999a) Lesson Study [jugyokenkyu] in elementary school mathematics in Japan: a case study. Paper presented at the American Educational Research Association annual meeting, Montreal, Canada

[CR33] Yoshida M (1999b) Lesson Study: a case study of a Japanese approach to improving instruction through school-based teacher development. (Dissertation), University of Chicago, Chicago

